# Effort-reward imbalance at work and the co-occurrence of lifestyle risk factors: cross-sectional survey in a sample of 36,127 public sector employees

**DOI:** 10.1186/1471-2458-6-24

**Published:** 2006-02-07

**Authors:** Anne Kouvonen, Mika Kivimäki, Marianna Virtanen, Tarja Heponiemi, Marko Elovainio, Jaana Pentti, Anne Linna, Jussi Vahtera

**Affiliations:** 1 Department of Psychology, POB 9, FIN-00014, University of Helsinki, Finland; 2 Finnish Institute of Occupational Health, Topeliuksenkatu 42 a A, FIN-00250 Helsinki, Finland; 3 National Research and Development Centre for Welfare and Health (STAKES), POB 220, FIN-00531 Helsinki, Finland; 4 Finnish Institute of Occupational Health, Hämeenkatu 10, FIN-20500 Turku, Finland

## Abstract

**Background:**

In occupational life, a mismatch between high expenditure of effort and receiving few rewards may promote the co-occurrence of lifestyle risk factors, however, there is insufficient evidence to support or refute this hypothesis. The aim of this study is to examine the extent to which the dimensions of the Effort-Reward Imbalance (ERI) model – effort, rewards and ERI – are associated with the co-occurrence of lifestyle risk factors.

**Methods:**

Based on data from the Finnish Public Sector Study, cross-sectional analyses were performed for 28,894 women and 7233 men. ERI was conceptualized as a ratio of effort and rewards. To control for individual differences in response styles, such as a personal disposition to answer negatively to questionnaires, occupational and organizational -level ecological ERI scores were constructed in addition to individual-level ERI scores. Risk factors included current smoking, heavy drinking, body mass index ≥25 kg/m^2^, and physical inactivity. Multinomial logistic regression models were used to estimate the likelihood of having one risk factor, two risk factors, and three or four risk factors. The associations between ERI and single risk factors were explored using binary logistic regression models.

**Results:**

After adjustment for age, socioeconomic position, marital status, and type of job contract, women and men with high ecological ERI were 40% more likely to have simultaneously ≥3 lifestyle risk factors (vs. 0 risk factors) compared with their counterparts with low ERI. When examined separately, both low ecological effort and low ecological rewards were also associated with an elevated prevalence of risk factor co-occurrence. The results obtained with the individual-level scores were in the same direction. The associations of ecological ERI with single risk factors were generally less marked than the associations with the co-occurrence of risk factors.

**Conclusion:**

This study suggests that a high ratio of occupational efforts relative to rewards may be associated with an elevated risk of having multiple lifestyle risk factors. However, an unexpected association between low effort and a higher likelihood of risk factor co-occurrence as well as the absence of data on overcommitment (and thereby a lack of full test of the ERI model) warrant caution in regard to the extent to which the entire ERI model is supported by our evidence.

## Background

The Effort-Reward Imbalance (ERI) model, a recent model of occupational stress, focuses on a negative trade-off between experienced 'costs' and 'gains' at work. In this model, high ratio of occupational effort spent relative to rewards received in turn in terms of money, esteem, job security, and career opportunities, elicits sustained stress responses and ill health [1-4]. This contractual reciprocity is frequent in cases where people have no alternative choice in the labor market or where they are exposed to heavy competition [5]. In addition to effort and rewards, the ERI model includes a third component, overcommitment, which refers to a set of attitudes, behaviours, and emotions reflecting excessive striving in combination with a strong desire to be approved of and esteemed. However, the evidence of adverse health effects is stronger for high efforts and low rewards (i.e., high ERI) than for overcommitment [6,7].

In addition to work-related morbidity, the model assumes that high ERI promotes lifestyle risk factors, such as smoking, high alcohol consumption, unhealthy dietary habits, and sedentary behavior. However, empirical research to support this hypothesis is scarce. There is some evidence suggesting a relation of high ERI or some of its components with smoking [8-10], alcohol consumption or dependence [11,12], and higher body mass index (BMI) [13].

Lifestyle risk factors tend to aggregate [14], and they may reinforce each other in their effects. The combined effects of adverse lifestyle factors have been demonstrated to be synergistic rather than additive [15]. No reports, to our knowledge, are available on the association between ERI and the co-occurrence of lifestyle risk factors.

We examined the relationship of ERI and its components to the co-occurrence of lifestyle risk factors in a sample of Finnish public sector employees. Our study aimed at adding to prior research in four ways. First, this is the first study to examine the ERI model in relation to the co-occurrence of lifestyle risk factors. Second, most previous studies have assessed ERI with individual-level self-reports. To control for individual differences in response styles, such as a personal disposition to answer negatively to questionnaires, we used occupational and organizational -level aggregated scores (in addition to individual-level scores) to model the effect of ERI. Aggregated scores were based on the responses of all the workers in the same occupation and organizational unit. Third, several third factors can confound associations in observational studies. We controlled for several confounders or possible predictors of risk factor co-occurrence, such as socioeconomic status (SES), age, marital status, and type of job contract (permanent vs. temporary). Fourth, very large data sets are required to examine co-occurring risk factors as co-occurrence of multiple risk factors is rare. To ensure statistical power, we focused on a large population covering over 36,000 employees in more than 1000 combinations of organizations and occupations. Indeed, this is one of the largest studies of the ERI model.

## Methods

### Study design and study sample

We used cross-sectional data from a large employee sample participating in an ongoing prospective Finnish Public Sector Study conducted in ten towns and 21 hospitals [16,17]. All workers employed in these organizations were invited to participate. Participation was voluntary. 39,255 women and 9337 men aged 17 to 65 were examined through self-administered questionnaires in 2000–2002. Response rate was 68% and the sample did not substantially differ from the eligible population. In the ten town sub sample, figures for participants vs. eligible population (N = 47,351) were as follows: mean age 44.9 vs. 44.5 years, proportion of women 77% vs. 72%, proportions of upper non-manual, lower non-manual and manual employees 34%, 46%, 20% vs. 35%, 42% and 22%, respectively. The corresponding figures for the hospital sub sample (N = 23,610) were: mean age 43.1 vs. 43.1 years, proportion of women 87% vs. 84%, proportions of upper non-manual, lower non-manual and manual employees 16%, 77%, 8% vs. 13%, 81% and 7%, respectively.

Respondents who did not provide information about all four lifestyle risk factors were excluded (N = 3636). We also excluded those with missing data on age (N = 27). Moreover, the survey instrument for the personnel of seven hospitals did not contain the ERI measure and there were also some other missing cases for ecological level ERI (N = 9911) and for the covariates (N = 1032). In consequence, the data set of the present study comprised 28,844 women and 7233 men who provided complete data with respect to age, ecological ERI, all four examined risk factors, and all covariates (74% of the respondents to the baseline survey).

In the Finnish Public Sector Study, written consent was obtained from the participants for linking register-based information on sickness absences with survey responses. Regarding the questionnaire survey (the present data), written consent was not obtained as the study was approved by the Ethics Committee of the Finnish Institute of Occupational Health.

### Measurements

The standard measure of ERI in Finnish was not available in this study. The questionnaire used included one question about effort in work and three questions about rewards. These measures were used to construct the proxy measure of ERI.

Effort in work was measured with the following question: "How much do you feel you invest in your job in terms of skill and energy?" Rewards were assessed with a scale containing three questions about feelings of getting in return from work in terms of (1) income and job benefits, (2) recognition and prestige, and (3) personal satisfaction (Cronbach's Alpha = 0.64) [10]. Response format for all the questions was a five-point Likert scale ranging from 1 = "very little" to 5 = "very much". Rewards were assessed as a mean score of the three rewards questions. If half or more of the component items were missing, a value of missing was recorded in the total reward score. To measure ERI, a ratio of effort (numerator) and the mean of rewards (denominator) was computed in accordance with the procedure used in recent publications [3,10,18].

The mean scores for effort, rewards and ERI were additionally calculated for each occupational group (Statistics Finland codes) within each workplace (town or hospital), a total of 1049 groups each including 10 or more employees. The mean scores (ecological scores) were applied to all members of the group. For both the individual-level measures and ecological measures, we divided the participants into tertiles according to the distribution of effort and rewards scores in the total study population. Similarly, we constructed tertiles of the effort-reward ratio to identify a high-risk group in terms of the upper tertile, while the lowest tertile indicated the most advantageous position of low effort relative to rewards.

Risk factors assessed in this study included current smoking, heavy drinking, being overweight as defined by body mass index (BMI) ≥25 kg/m^2 ^[19], and physical inactivity. The four risk factors were dichotomized as to be adherent to the public health recommendation (no risk referred to non-smoking, non- or moderate drinking, BMI <25 kg/m^2^, and physical activity ≥2 metabolic equivalent task hours per day).

Smoking was assessed by a question on whether the respondent was a current smoker or not. The respondents reported their habitual frequency and amount of beer, wine, and spirits intake. One unit of pure alcohol (12 g) is equal to a 12-cl glass of wine, a single 4-cl measure of spirits, or a 33-cl bottle of beer. A dichotomous variable was created to represent heavy drinking, with a cut-off point corresponding to an average weekly consumption ≥190 g [20] of absolute alcohol for women and >275 g for men [21].

Participants reported the average amount of time spent per week on leisure and on the journey to and from work in physical activity corresponding to the activity intensity of walking, vigorous walking, jogging, and running. The time spent at each activity in hours per week was multiplied by its typical energy expenditure, expressed in metabolic equivalent tasks (METs). Activity MET index was expressed as the summary score of MET-hours/week. Participants whose volume of activity was < 2 MET-hours/day were classified as being physically inactive [22].

Co-occurrence of lifestyle risk factors was defined as the number of risk factors for which an individual participant in question had high-risk values. If a participant belonged to the high risk group for all four risk factors, the corresponding co-occurrence score was 4; if the participant belonged to three "high risk" groups, the co-occurrence score was 3, etc. Since the number of employees having all four risk factors was very low [0.3% (N = 98) of the women and 1% (N = 96) of the men], we collapsed the co-occurrence score into four categories, ranging from 0 (having none of the four risk factors) to 3 (having three or four risk factors).

Potential confounders measured were: sex, age group (17–34, 35–50, and 51–63 years), marital status (married or cohabiting vs. single, divorced, or widowed), socioeconomic position, and type of job contract (permanent vs. fixed term). Information on sex, age, occupational title, and type of job contract was obtained from the employers' records. The occupational titles, expressed as five-digit codes of Statistics Finland, were categorized into the socioeconomic positions of manual, and lower and upper non-manual work following the Statistics Finland classification.

### Statistical analysis

Multinomial logistic regression models were calculated to investigate the relationship of ecological and individual level effort, rewards, and ERI with the co-occurrence of lifestyle risk factors. Participants with one, two, and ≥3 risk factors were compared with those with no risk factors. We used the employees in the most favorable tertiles for each of the ERI dimensions as reference groups.

All models were fit separately for women and men. Due to different cut-off points of heavy drinking, exact numerical comparison of the results between women and men is not possible. The analyses were made in two phases. Firstly, the associations were examined adjusting only for age. Then, marital status, socioeconomic position, and type of job contract were added to the model as covariates to see how this affected the associations. To further test the association and to take into account the fact that individual employees were nested within work units, we performed the logistic regression analysis with generalized estimating equations (GEE) method [23] comparing those with ≥3 risk factors with those with 0 to 2 risk factors.

Binary logistic regression analyses were performed to examine the associations between ecological ERI and the likelihood of single risk factors.

The results are presented as odds ratios (ORs) and their 95% confidence intervals (CIs). Data were examined with SPSS for Windows 12.0.1 (SPSS, Inc., Chicago, Illinois) and SAS V8 (SAS Institute, Cary, NC) program packages.

## Results

### Participant characteristics

The mean number of lifestyle risk factors by socio-demographic variables, and the prevalence of single risk factors are presented in Table 1. The mean number of risk factors was 0.9 (SD = 0.9) for the women and 1.2 (SD = 0.9) for the men. Four percent of the women and 9% of the men had ≥3 risk factors. The proportion of women with none of the four risk factors was 38%, and the corresponding figure for the men was 24%. In both women and men, the mean number of risk factors was significantly higher among older people, manual workers, permanent employees, and among participants living without a partner. (p < 0.001 in all cases.)

### Association between ERI and the co-occurrence of risk factors

Adjusted ORs (95% CIs) for co-occurring risk factors among the women are presented in Table 2. Exposure to high ecological ERI was associated with a higher likelihood of risk factor co-occurrence, and the excess risk persisted although lowered after adjustment for marital status, socioeconomic position, and type of job contract. In the fully adjusted model, the women with high ecological ERI (OR = 1.44, 95% CI 1.23–1.69) were more likely than their counterparts with low ERI to have ≥3 risk factors (vs. 0 risk factors). When examined separately, both components of ecological ERI, low effort and low rewards, were also associated with a higher likelihood of risk factor co-occurrence. The analyses using individual-level measures of ERI largely replicated the results.

Table 3 shows that the results for the men were mostly in the same direction as those for the women. In the fully adjusted model men with the greatest disparity between ecological effort and rewards had a 1.4-fold odds for ≥3 risk factors (vs. 0 risk factors) compared with the men with low ecological ERI. Low effort was statistically significantly associated with the co-occurrence of ≥3 risk factors before but not after adjustment for all of the covariates. However, low ecological effort was associated with the co-occurrence of 2 risk factors also in the fully adjusted model. Low ecological rewards were associated with the co-occurrence of risk factors irrespective of adjustments. The analyses using individual measures of ERI largely replicated these results. However, the association between low effort and a higher likelihood of the co-occurrence of ≥3 risk factors (vs. 0 risk factors) remained significant in the fully adjusted model.

With the exception of ecological effort and individual rewards in the men, among both the women and the men the associations for the comparisons of 1 vs. 0 risk factors and 2 vs. 0 risk factors were weaker than the associations for the comparisons of ≥3 vs. 0 risk factors.

The results of logistic regression analyses with GEE method were in the same direction as the results presented here suggesting that the hierarchical structure of the data was an unlikely source of major bias in this study.

### Association between ERI and single risk factors

Table 4 presents the associations between ecological ERI and single dichotomous risk factors. After adjustment for age, marital status, socioeconomic position, and type of job contract, high ERI was associated with a higher likelihood of smoking (OR = 1.45, 95% CI: 1.33–1.58), physical inactivity (OR = 1.07, 95% CI: 1.00–1.15), and BMI ≥25 kg/m^2 ^(OR = 1.08, 95% CI: 1.02–1.15) among the women. Although high ERI was also related to these risk factors among the men in the age adjusted models, a significant association was detected only for physical inactivity (OR = 1.23, 95% CI: 1.07–1.41) in the fully adjusted models. Of the ERI components, lower effort and lower reward were associated with a higher likelihood of smoking among the women. Moreover, among both sexes, lower effort was associated with a higher likelihood of physical inactivity and being overweighted and lower rewards were associated with a higher likelihood of physical inactivity.

## Discussion

According to the effort-reward imbalance model, high effort-reward imbalance would be expected to be associated with a higher likelihood of the lifestyle risk factor co-occurrence. The present results from a sample of 36,127 Finnish employees gave moderate support for this hypothesis. High ERI at the ecological occupational and organizational level was associated with 40% higher odds of having ≥3 lifestyle risk factors (vs. 0 risk factors) as compared with jobs with low ERI. The results with the individual level scores were in the same direction than those obtained with the ecological scores.

When the ecological ERI components were examined separately, low rewards and low effort were associated with an elevated prevalence of risk factor co-occurrence. The first finding was in line with the hypothesis, whereas the latter result was unexpected as according to the theory of ERI, low effort should be associated with a lower rather than a higher likelihood of risk factor co-occurrence. As a result, the ERI model was only partially supported by our findings. Furthermore, given the unexpected findings for high efforts, the adverse effects of the total ERI measure may solely be due to the adverse effects of low rewards.

The measurement of effort with a single item not included in the standard assessment instrument may have contributed to an unexpected finding. Further research should preferable use the original ERI measures for efforts. Moreover, in our data, effort and rewards were significantly positively correlated (*r *= .24, p < 0.001). Low effort might represent passive life orientation or lifestyle, which was manifested also with single risk factors: in our data, low effort was associated with a higher likelihood of sedentary lifestyle and being overweight. If our measurement of high effort tapped active lifestyle rather than work overload, then these results should not be interpreted as counterevidence for the ERI model.

The associations between ERI, its components and co-occurring risk factors were independent of individual-level confounders such as age, marital status, socioeconomic position, and type of job contract. Therefore, although the possibility of confounding by an unknown factor can never be totally excluded, a major bias in our study is unlikely. However, compared with the age adjusted model, the association weakened after adjustments and further analysis showed that the attenuation was mostly due to socioeconomic position. The effects of socio-economic and psychosocial factors are often difficult to separate. Rewards gained from work highly differ between socioeconomic groups. Employees with lower socioeconomic position typically report higher ERI at work and they suffer more from the adverse consequences of ERI with respect to cardiovascular risk [4]. Including socioeconomic position as a covariate in the model when studying the health effects of ERI may thus lead to an over-adjustment.

This is apparently the first study to show that high ERI at work is associated with the co-occurrence of multiple lifestyle risk factors. Our findings rely on a large survey with a reasonable response rate. The respondents represented the target population satisfactorily in terms of mean age and the distribution by socioeconomic position. Demographic information obtained from the employers' registers was available to practically all participants. Findings were replicated using both individual and ecological measures of ERI. In cross-sectional data common method variance may artificially inflate relationships between variables and may bias the results concerning bivariate associations [24]. By using ecological approach, which is only possible in such large data sets as ours, problems related to common-method variance were largely avoided.

High cost – low gain conditions at work, such as having a demanding but an unstable job, or achieving at a high level without being offered any promotion prospects [2] could generate feelings of frustration, negative attitudes toward work, low job satisfaction, as well as general passivity and apathy. The alienation from work could be associated with adoption of unhealthy behaviors [25]. It is possible that some workers with jobs and workplaces characterized by high ERI use unhealthy behaviors as a response for their unsatisfactory job conditions. For example, they may eat for comfort [26] and use smoking and excess drinking as a means of coping [27]. In addition, emotional stress may be an obstacle to initiate or maintain exercise behavior [28], or it may postpone decisions to quit smoking. Earlier research has indicated an association of mental distress [29] and depression [14] with the number of lifestyle risk factors. In a similar way, ERI has been associated with depression [30]. These and similar factors can potentially be among the mechanisms linking ERI with co-occurrence of lifestyle risk factors.

Earlier evidence, which is scarce and which relates to single risk factors, is characterized by relatively weak associations. In the present study, the associations of ecological ERI with single risk factors were less marked than those with the co-occurrence of risk factors with the exception that in women high ERI and low rewards were associated with current smoking as strongly as with the co-occurrence of ≥3 risk factors and among the men low rewards were more strongly associated with physical inactivity than with the co-occurrence of ≥3 risk factors.

There may be individual differences in behavioral reactions to stress, some people eating more and gaining weight during stress, some smoking more intensively when suffering from stress and some people becoming physically inactive during stressful periods of life. For this reason, the associations of ERI with specific risk behaviors are expected to be relatively weak, whereas the associations of ERI with having one or some of the risk factors (irrespective of their combination) are expected to be stronger. Risk factors tend to cluster in same individuals and the novel finding of this study is that high ERI may increase the likelihood of such a clustering. This finding emphasizes the importance of focusing on the co-existence of risk factors. Moreover, it has been shown that the co-occurrence of multiple lifestyle risk factors greatly influences morbidity and mortality and that the effects of negative health practices are cumulative. For example, Meng et al. [15] detected a positive synergistic effect between smoking and BMI in regard to mortality: whereas the sum of the excess risk ratios of current smoking and BMI for male total mortality was only 1.3, but the observed combined risk ratio was 2.4.

### Limitations of the study

We used a large-scale sample, but the study was based on a cross-sectional design. Therefore we do not claim that the observed associations are evidence of a causal relationship. Although high ERI may lead to an increased likelihood of the co-occurrence of lifestyle risk factors, such unsatisfactory job conditions may sometimes also reflect these adverse behaviors, either as the effects of behaviors itself or as the effects of being a smoking, heavily drinking, overweight, and physically inactive employee. Workers with multiple unhealthy behaviors may not invest as much on their work and career than their healthy-living counterparts. Unhealthy lifestyle and poor health may lead to downward drift to low status jobs with high contractual non-reciprocity and job insecurity. However, that is a less likely explanation for the results based on ERI indicator derived from ecological scores.

The original ERI measure was not available in this study. In particular, our measure did not include overcommitment, which refers to a personal pattern of coping with work demands – excessive striving in combination with a strong desire to be approved of and esteemed. Overcommitment is hypothesized to amplify the adverse health effects produced by ERI, because overcommitted workers exaggerate their efforts beyond the normally considered appropriate [1,3].

However, the study by Fahlen et al. [31] showed that the approximate and the original ERI instrument pointed to the same direction and in general both studies using original and proxy measures have found support for the ERI model, indicating an effect of ERI regardless of the measure being used [6]. Furthermore, previous reports of this study cohort have shown an association between high ERI and increased body mass index [13] and smoking intensity [10], an indication of the predictive validity of our ERI measure. In spite of this, there is a possibility that our measure did not fully capture the ERI model and the Cronbach's Alpha for the rewards scale was moderate. These issues may have underestimated the associations observed. Further research with original ERI measures is therefore needed to confirm the present findings.

The magnitude of the associations was rather small. However, this study was not based on the assumption that ERI is the major determinant of lifestyle-related risk factors and their co-occurrence, but we rather hypothesized that ERI might be one of the factors influencing these behaviors [32]. Besides, cumulative or chronic ERI over a long period of time is associated with higher risk of disease compared to single assessment [33].

Although the use of aggregated data reduced problems related to common method variance and response bias, this ecological approach has a potential weakness. In aggregated scores the subjective component is largely excluded, and it is possible that the essence of the ERI model is just this subjective component, i.e., only perception of ERI is likely to elicit an adverse effect on health and health behaviors. The individual-level analysis might better capture that perceptual component and thus ecological analysis is likely to provide a conservative estimate of the true association between ERI and co-occurring risk factors.

The dichotomization of risk factors enabled assessment of co-occurring risk factors but may have reduced statistical power thereby underestimating the strength of the associations. If we had chosen lower cut-off points, the proportion of high-risk individuals would have increased notably. Moreover, the self-report nature of the data makes them subject to recall and response bias. Nonresponse and misclassification are likely to influence different behaviors to differing degrees. For example, self-reported current smoking is probably more precise than self-reported alcohol use [34].

Although the sample size was large, the present data were female-dominated and from the public sector. The respondents were representative of Finnish public sector employees in terms of sex and age, but the female predominance did not correspond to the sex distribution of the Finnish general working population. Therefore, the findings should be interpreted with caution until they are validated in studies using other samples.

Finally, we did not have sufficient information to include all important lifestyle risk factors. In particular, information on dietary habits was lacking. However, since BMI is influenced by energy intake (as well as physical activity), inclusion of it has probably partially accounted for dietary habits.

## Conclusion

To summarize, findings from a large public sector employee population indicate that failed reciprocity in work may be associated with the co-occurrence of the most important potentially preventable lifestyle-related risk factors that contribute to cardiovascular disease and other chronic diseases [35]. However, an unexpected association between low effort and a higher likelihood of risk factor co-occurrence as well as the absence of data on overcommitment (and thereby a lack of full test of the ERI model) warrant caution in regard to the extent to which the entire ERI model is supported by our evidence.

If confirmed by prospective studies with other study populations, this moderately supportive evidence implies that the reduction of effort-reward imbalance at work could help efforts to improve health behaviors among working population.

## List of abbreviations

BMI = body mass index; ERI = effort-reward imbalance MET = metabolic equivalent task; OR = odds ratio.

## Competing interests

The author(s) declare that they have no competing interests.

## Authors' contributions

AK designed the study, carried out the data analyses and was the principal author of the paper. MK and JV are the directors of the Finnish Public Sector Study and the second principal investigators of the study; they helped in designing the study, the data analysis, interpreting the results, and writing the paper. MV and AL collected the data, contributed to interpretation of the results, and edited the manuscript. JP constructed the ecological measures and supervised the data analyses. TH and ME contributed to interpretation of the results and manuscript writing.

All authors read and approved the final manuscript.

## Pre-publication history

The pre-publication history for this paper can be accessed here:



## Figures and Tables

**Table 1:** Descriptive statistics and the mean number of risk factors by sociodemographic variables and Effort-Reward Imbalance (ERI)

	**Women (N = 28,894)**		**Men (N = 7233)**	
		
	**N (%)**	**Mean number of risk factors (SD)**	**N (%)**	**Mean number of risk factors (SD)**
**Age group (yr)**				
17 to 34	4913 (17)	0.7 (0.8)	1181 (16)	1.0 (0.9)
35 to 50	14,878 (51)	0.9 (0.9)	3543 (49)	1.2 (0.9)
51 to 64	9103 (32)	1.0 (0.9)	2509 (35)	1.3 (0.9)
		p < 0.001		p < 0.001
				
**Marital status**				
Married or cohabiting	21,506 (75)	0.9 (0.8)	5834 (81)	1.2 (0.9)
Single, divorced or widowed	7311 (25)	0.9 (0.9)	1399 (19)	1.3 (1.0)
		p < 0.001		p < 0.001
				
**Socioeconomic position**				
Manual	3579 (12)	1.1 (0.9)	2404 (33)	1.4 (0.9)
Lower non-manual	17,119 (59)	0.9 (0.8)	2057 (28)	1.2 (0.9)
Upper non-manual	8196 (28)	0.8 (0.8)	2767 (38)	1.0 (0.9)
		p < 0.001		p < 0.001
				
**Type of job contract**				
Permanent	23,833 (83)	0.9 (0.9)	6282 (87)	1.2 (0.9)
Fixed term	5061 (18)	0.8 (0.8)	951 (13)	1.0 (0.9)
		p < 0.001		p < 0.001
				
**Effort-Reward Imbalance (ecological score)**				
Low	9291 (32)	0.8 (0.8)	2826 (39)	1.1 (0.9)
Intermediate	10,202 (35)	0.8 (0.8)	2059 (28)	1.2 (0.9)
High	9710 (33)	1.0 (0.9)	2417 (33)	1.4 (0.9)
		p < 0.001		p < 0.001
				
**Effort-Reward Imbalance (individual score)**				
Low	9001 (31)	0.9 (0.8)	2657 (37)	1.2 (0.9)
Intermediate	11,085 (38)	0.9 (0.8)	2429 (33)	1.2 (0.9)
High	8758 (30)	0.9 (0.9)	2151 (30)	1.3 (1.0)
		p < 0.001		p < 0.001
				
**Lifestyle risk factors**				
Current smoker	4978 (17)		1715 (24)	
Heavy drinker*	2210 (8)		890 (12)	
Physically inactive^†^	7065 (25)		1950 (27)	
Body mass index ≥25 kg/m^2^	11,454 (40)		4252 (59)	
				
**No. of risk factors**				
Zero	10,958 (38)		1731 (24)	
One	11,437 (40)		2918 (40)	
Two	5323 (19)		19537(27)	
Three or four	1176 (4)		647 (9)	

Only participants with no missing data in any of the variables were included. (In analyses for the individual level ERI score, N = 28,544 for women and N = 7170 for men.)

SD, standard deviation

p values from Chi Square test.

*Average weekly consumption ≥190 g of absolute alcohol for women and >275 g for men.

^†^< 2 MET hours.

**Table 2:** Associations between Effort-Reward Imbalance (ERI) dimensions and co-occurrence of risk factors in women (N = 28,894)

	**Age adjusted model**			Fully adjusted model*		
		
	**1 vs 0 risk factors**	**2 vs 0 risk factors**	**3–4 vs 0 risk factors**	**1 vs 0 risk factors**	**2 vs 0 risk factors**	**3–4 vs 0 risk factors**
	**OR (95% CI)**^†^	**OR (95% CI)**	**OR (95% CI)**	**OR (95% CI)**	**OR (95% CI)**	**OR (95% CI)**
**Ecological score**						
Effort (component of ERI)						
Low	1.00	1.00	1.00	1.00	1.00	1.00
Intermediate	**0.88 **(0.82–0.94)	**0.82 **(0.75–0.89)	**0.78 **(0.67–0.90)	0.96 (0.90–1.03)	0.93 (0.86–1.01)	0.93 (0.80–1.09)
High	**0.79 **(0.74–0.84)	**0.65 **(0.60–0.70)	**0.59 **(0.51–0.69)	0.94 (0.87–1.01)	**0.81 **(0.74–0.89)	**0.80 **(0.67–0.95)
Rewards (component of ERI)						
High	1.00	1.00	1.00	1.00	1.00	1.00
Intermediate	1.05 (0.98–1.11)	**1.12 **(1.03–1.21)	1.08 (0.92–1.26)	0.97 (0.95–1.11)	1.03 (0.94–1.12)	0.98 (0.84–1.16)
Low	**1.26 **(1.18–1.35)	**1.61 **(1.48–1.75)	**1.90 **(1.63–2.20)	1.03 (0.95–1.11)	**1.22 **(1.11–1.35)	**1.35 **(1.13–1.60)
Effort-Reward Imbalance						
Low ERI	1.00	1.00	1.00	1.00	1.00	1.00
Intermediate ERI	1.00 (0.93–1.06)	**1.10 **(1.01–1.19)	1.05 (0.89–1.23)	0.98 (0.92–1.05)	1.07 (0.99–1.16)	1.02 (0.87–1.19)
High ERI	**1.21 **(1.13–1.29)	**1.51 **(1.39–1.64)	**1.83 **(1.57–2.12)	**1.07 **(1.00–1.15)	**1.25 **(1.15–1.37)	**1.44 **(1.23–1.69)
						
**Individual score**						
Effort (component of ERI)						
Low	1.00	1.00	1.00	1.00	1.00	1.00
Intermediate	**0.87 **(0.80–0.95)	**0.78 **(0.69–0.86)	**0.72 **(0.60–0.87)	**0.91 **(0.84–1.00)	**0.84 **(0.77–0.96)	**0.80 **(0.67–0.97)
High	**0.86 **(0.78–0.94)	**0.77 **(0.70–0.87)	**0.70 **(0.58–0.86)	0.93 (0.85–1.02)	**0.86 **(0.77–0.96)	**0.82 **(0.67–1.00)
Rewards (component of ERI)						
High	1.00	1.00	1.00	1.00	1.00	1.00
Intermediate	**1.09 **(1.03–1.16)	**1.11 **(1.03–1.20)	1.13 (0.97–1.31)	**1.07 **(1.00–1.14)	1.08 (1.00–1.16)	**1.09 **(0.93–1.27)
Low	**1.17 **(1.10–1.26)	**1.33 **(1.23–1.45)	**1.69 **(1.45–1.96)	**1.11 **(1.04–1.19)	**1.22 **(1.12–1.33)	**1.51 **(1.30–1.76)
Effort-Reward Imbalance						
Low ERI	1.00	1.00	1.00	1.00	1.00	1.00
Intermediate ERI	1.01 (0.95–1.07)	0.99 (0.91–1.07)	0.98 (0.84–1.14)	1.02 (0.96–1.09)	1.01 (0.93–1.09)	1.01 (0.87–1.17)
High ERI	**1.10 **(1.02–1.17)	**1.19 **(1.10–1.30)	**1.36 **(1.17–1.59)	**1.07 **(1.00–1.15)	**1.15 **(1.06–1.26)	**1.31 **(1.12–1.52)

Only participants with no missing data in any of the covariates or ecological ERI were included in these models. (In the analyses for the individual ERI score, N = 28,544.)

ERI, Effort-Reward Imbalance; OR, odds ratio; CI, confidence interval

*Adjusted for age, socioeconomic position, job contract, and marital status.

^†^Statistically significant at 95% confidence level or better bolded.

Risk factors are current smoker, BMI ≥25 kg/m^2^, physically inactive, and heavy drinker, where inactive individuals have <2 MET-hours/day, and heavy drinkers are women who consume on average >190 g of absolute alcohol per week.

**Table 3:** Associations between Effort-Reward Imbalance (ERI) dimensions and co-occurrence of risk factors in men (N = 7233)

	**Age adjusted model**			Fully adjusted model*		
		
	**1 vs 0 risk factors**	**2 vs 0 risk factors**	**3–4 vs 0 risk factors**	**1 vs 0 risk factors**	**2 vs 0 risk factors**	**3–4 vs 0 risk factors**
	**OR^† ^(95% CI)**	**OR (95% CI)**	**OR (95% CI)**	**OR (95% CI)**	**OR (95% CI)**	**OR (95% CI)**
**Ecological score**						
Effort (component of ERI)						
Low	1.00	1.00	1.00	1.00	1.00	1.00
Intermediate	**0.77 **(0.65–0.92)	**0.57 **(0.47–0.68)	**0.47 **(0.36–0.62)	1.00 (0.82–1.23)	0.86 (0.69–1.08)	0.80 (0.58–1.11)
High	**0.60 **(0.52–0.68)	**0.43 **(0.37–0.49)	**0.36 **(0.29–0.45)	0.88 (0.72–1.08)	**0.75 **(0.60–0.95)	0.78 (0.56–1.08)
Rewards (component of ERI)						
High	1.00	1.00	1.00	1.00	1.00	1.00
Intermediate	**1.50 **(1.27–1.78)	**1.65 **(1.37–1.99)	**1.65 **(1.24–2.18)	1.15 (0.95–1.39)	1.24 (1.00–1.53)	1.15 (0.84–1.58)
Low	**1.55 **(1.36–1.77)	**2.35 **(2.03–2.72)	**2.95 **(2.39–3.64)	0.96 (0.78–1.18)	**1.30 **(1.04–1.63)	**1.46 **(1.06–2.01)
Effort-Reward Imbalance						
Low ERI	1.00	1.00	1.00	1.00	1.00	1.00
Intermediate ERI	1.05 (0.91–1.21)	1.06 (0.91–1.25)	1.11 (0.88–1.40)	0.99 (0.86–1.15)	0.99 (0.84–1.16)	1.00 (0.79–1.26)
High ERI	**1.41 **(1.22–1.64)	**1.93 **(1.64–2.25)	**2.32 **(1.87–2.88)	1.06 (0.89–1.25)	**1.22 **(1.01–1.46)	**1.36 **(1.06–1.74)
						
**Individual score**						
Effort (component of ERI):						
Low	1.00	1.00	1.00	1.00	1.00	1.00
Intermediate	0.85 (0.72–1.01)	**0.72 **(0.60–0.85)	**0.60 **(0.48–0.76)	0.95 (0.80–1.12)	0.85 (0.71–1.02)	**0.75 **(0.60–0.95)
High	**0.80 **(0.67–0.96)	**0.60 **(0.50–0.74)	**0.44 **(0.34–0.58)	0.96 (0.80–1.16)	**0.81 **(0.66–1.00)	**0.64 **(0.48–0.85)
Rewards (component of ERI)						
High	1.00	1.00	1.00	1.00	1.00	1.00
Intermediate	1.13 (0.98–1.31)	**1.35 **(1.15–1.58)	**1.30 **(1.02–1.64)	1.03 (0.89–1.19)	1.16 (0.98–1.37)	1.06 (0.84–1.35)
Low	**1.36 **(1.17–1.57)	**1.94 **(1.65–2.28)	**2.31 **(1.85–2.89)	1.13 (0.97–1.32)	**1.46 **(1.23–1.73)	**1.61 **(1.27–2.04)
Effort-Reward Imbalance						
Low ERI	1.00	1.00	1.00	1.00	1.00	1.00
Intermediate ERI	1.07 (0.93–1.23)	1.02 (0.87–1.19)	0.84 (0.67–1.06)	1.05 (0.91–1.21)	1.00 (0.85–1.17)	0.82 (0.66–1.03)
High ERI	**1.23 **(1.06–1.43)	**1.45 **(1.23–1.71)	**1.53 **(1.23–1.91)	1.12 (0.96–1.31)	**1.26 **(1.07–1.49)	**1.29 **(1.03–1.61)

Only participants with no missing data in any of the covariates or ecological ERI were included in these models. (In the analyses for the individual ERI score, N = 7170.)

OR, odds ratio; CI, confidence interval

* Adjusted for age, socioeconomic position, job contract, and marital status.

^†^Statistically significant at 95% confidence level or better bolded.

Risk factors are current smoker, BMI ≥25 kg/m^2^, physically inactive, and heavy drinker, where inactive individuals have <2 MET-hours/day, and heavy drinkers are men who consume on average ≥275 g of absolute alcohol per week.

**Table 4:** Associations between ecological Effort-Reward Imbalance (ERI) dimensions and single risk factors (adjusted* odds ratios, 95% confidence intervals)

	**Smoking**	**Heavy drinking**^†^	**Physical inactivity ‡**	**BMI ≥25 kg/m**^2^
				
**Women (N = 28,894):**				
Effort (component of ERI)				
Low	1.00	1.00	1.00	1.00
Intermediate	1.01 (0.93–1.09)	1.00 (0.88–1.13)	**0.91 **(0.85–0.98)	0.99 (0.93–1.05)
High	**0.89 **(0.81–0.97)	1.11 (0.98–1.27)	**0.82 **(0.76–0.88)	**0.93 **(0.87–0.99)
Rewards (component of ERI)				
High	1.00	1.00	1.00	1.00
Intermediate	**1.17 **(1.08–1.28)	**0.88 **(0.79–0.98)	1.01 (0.94–1.08)	0.96 (0.90–1.02)
Low	**1.42 **(1.30–1.56)	0.92 (0.82–1.05)	**1.12 **(1.03–1.21)	1.03 (0.96–1.11)
Effort-Reward Imbalance				
Low ERI	1.00	1.00	1.00	1.00
Intermediate ERI	**1.21 **(1.11–1.31)	1.05 (0.94–1.16)	0.96 (0.89–1.02)	0.98 (0.92–1.04)
High ERI	**1.45 **(1.33–1.58)	0.99 (0.88–1.11)	**1.07 **(1.00–1.15)	**1.08 **(1.02–1.15)
**Men (N = 7233):**				
Effort (component of ERI)				
Low	1.00	1.00	1.00	1.00
Intermediate	0.97 (0.81–1.17)	0.96 (0.75–1.23)	**0.80 **(0.67–0.96)	**0.84 **(0.71–0.99)
High	0.92 (0.75–1.12)	1.05 (0.82–1.34)	**0.81 **(0.67–0.97)	0.96 (0.82–1.13)
Rewards (component of ERI)				
High	1.00	1.00	1.00	1.00
Intermediate	0.97 (0.80–1.17)	1.04 (0.82–1.31)	1.16 (0.97–1.38)	1.09 (0.93–1.29)
Low	1.13 (0.93–1.37)	1.12 (0.88–1.43)	**1.43 **(1.19–1.72)	1.12 (0.96–1.31)
Effort-Reward Imbalance				
Low ERI	1.00	1.00	1.00	1.00
Intermediate ERI	0.93 (0.80–1.07)	1.10 (0.93–1.32)	1.01 (0.88–1.15)	1.00 (0.89–1.13)
High ERI	1.13 (0.98–1.30)	1.08 (0.89–1.31)	**1.23 **(1.07–1.41)	1.08 (0.95–1.23)

Only participants with no missing data in any of the covariates, risk factors or ecological ERI were included in these models.

* Adjusted for age, socioeconomic position, job contract, and marital status. Statistically significant at 95% confidence level or better bolded.

^† ^Average weekly consumption ≥190 g of absolute alcohol for women and >275 g for men.

‡ <2 MET-hours/day
